# Non-adherence to COVID-19 containment behaviours: results from an all-Ireland telephone survey

**DOI:** 10.1186/s12889-022-13322-6

**Published:** 2022-05-05

**Authors:** Martin Dempster, Nicola O’Connell, Christopher D. Graham, Cliodhna O’Connor, Lina Zgaga, Emma Burke, Luke Mather, Gail Nicolson, Joe Barry, Gabriel Scally, Ann Nolan, Katy Tobin, Philip Crowley, Catherine D. Darker

**Affiliations:** 1 School of Psychology, Queen’s University Belfast, 18-30 Malone Road, Belfast, BT9 5BN UK; 2 Discipline of Public Health and Primary Care, Institute of Population Health, Trinity College Dublin, Tallaght Cross, Dublin, D24 DH74 Ireland; 3 School of Medicine, University of Bristol, Tyndall Venue, Bristol, BS8 1TH UK; 4 Trinity Centre for Global Health, Trinity College Dublin, 7-9 Leinster Street South, Dublin, D02 K104 Ireland; 5 School of Medicine, Global Brain Health Institute, Trinity College Dublin, Lloyd Building, Dublin, D02 PN40 Ireland; 6 Quality Improvement, Health Service Executive, Dr Steevens’ Hospital, Dublin, D08 W2A8 Ireland

**Keywords:** COVID-19, Non-adherence, Public health messaging, Handwashing, Social distancing, Public health guidelines, Protection motivation theory

## Abstract

**Background:**

COVID-19 public health measures like handwashing and social distancing can help stem the spread of the virus. Adherence to guidelines varies between individuals. This study aims to identify predictors of non-adherence to social distancing and handwashing guidelines.

**Methods:**

A cross-sectional weekly telephone survey was conducted over eight weeks (11/06/2020–05/08/2020). The sample included adults resident on the island of Ireland (75:25 split between ROI and NI). Data were collected on demographics, threat perceptions, fear of COVID-19, response efficacy and self-efficacy, response cost and social norms, COVID-19 behaviours, mood, loneliness, and self-reported health.

**Results:**

3011 participants were surveyed. Handwashing non-adherers were more likely to be male (OR: 5.2, 95% CI: 2.4 – 11.3), to have higher levels of loneliness (OR: 1.86, 95% CI: 1.1 – 3.1), and higher perceptions of handwashing costs (OR: 3.4, 95% CI: 2.2 – 5.2). Those reporting rarely engaging in social distancing were more likely to be members of lower socioeconomic groups, to be younger (OR: 0.97, 95% CI: 0.96 – 0.98), male (OR: 1.67, 95% CI: 1.1 – 2.5), healthcare workers (OR: 1.98, 95% CI: 1.1 – 3.4), to report lower mood (OR: 1.72, 95% CI: 1.3 – 2.2), were less likely to live in households with people aged under-18 (OR: 0.75, 95% CI: 0.6 – 0.9), and to have lower fear of COVID-19 (OR: 0.79, 95% CI: 0.6 – 0.9).

**Conclusions:**

Non-adherers to handwashing differ to social distancing non-adherers. Public health messages should target specific demographic groups and different messages are necessary to improve adherence to each behaviour.

## Background

The novel coronavirus (SARS-CoV2), COVID-19 was first reported in the city of Wuhan, China in December 2019 [1]. With no widespread pharmacological interventions routinely available, public health measures such as physical distancing, handwashing and face masks were enacted to mitigate the spread of the virus.

Evidence from previous epidemics suggests that there is variation in the extent to which people adhere to these public health measures and there are a range of variables that can help to explain this variation [2–6].) Protection Motivation Theory (PMT) is a health behaviour model that summarises some of the key variables that have been shown to explain variations in motivation to engage in protective behaviours in the face of health threats [7]. PMT posits that motivations to protect ourselves from perceived threats are determined by threat and coping appraisals [8]. Threat appraisals comprise individuals’ judgements of the level of threat and estimations of vulnerability to this threat. Coping appraisals comprise self-efficacy beliefs (belief in own ability to engage in the protective behaviour), response efficacy (belief that the behaviour will mitigate the threat), and response costs (estimations of the cost of the protective behaviour to the individual).

PMT has successfully predicted behaviours such as vaccination uptake [9], nutrition intake [10], and smoking habits [11] and can help guide health communication strategies [12]. PMT is particularly relevant to COVID-19 as it can help explain the differential adoption of protective behaviours amongst groups and jurisdictions. For example, previous research has found positive correlations between preventative COVID-19 behaviour and perceived vulnerability, severity, response efficacy, protection motivation and self-efficacy [13–15]. Better understanding of the link between PMT components and non-adherence to COVID-19 preventive behaviours, could help explain these behaviours.

The objective of this study is to apply PMT to better understand individual variation in adherence to COVID-19 protective behaviours in the general public on the island of Ireland, and to make recommendations on effective targeting of public health messaging. The island of Ireland includes two governmental and public health jurisdictions – Northern Ireland (NI) and the Republic of Ireland (ROI) and responses may vary by health guidelines. The first COVID-19 cases were announced in NI on 27^th^ February and on 29th February in the ROI. Previous evidence suggests social norms can predict health behaviours, so we include this variable in our model [16]. Previous PMT studies examining COVID-19 protective behaviour have used small sample sizes [17] and non-representative convenience samples of participants recruited online who are often younger than one would expect from a representative sample [13, 14, 18].

The objective of our research is to better target subsequent public health messaging. This study forms part of a larger project which examines psychological, media, social media and policy responses to the COVID-19 pandemic on the island of Ireland [19].

The current study has four aims:

1. Apply PMT and social norm variables to delineate common drivers of non-adherence to COVID-19 protective behaviours (self-reported social distancing and handwashing).
2. Assess whether there are differences in the profile of people reporting non-adherence with handwashing and social distancing.
3. Examine the relationship between non-adherence to COVID-19 protective behaviours and components of well-being (mood and loneliness).
4. Compare protective behaviours and PMT variables in respondents living in NI and ROI.

## Methods

### Study design and participants

A detailed study protocol is available [19]. The research team commissioned Ipsos-MRBI to conduct an all-Ireland telephone survey, using a random digit dialling sampling strategy (80% landline, 20% mobile phone) on a weekly basis over eight weeks (11/06/2020–05/08/2020). It was cross-sectional, with new respondents each week. Eligibility criteria included individuals aged 18 years or over; the ability to communicate in English; possession of a landline or mobile phone; and current residency in ROI or NI.

A 75:25 sampling split was applied to ROI and NI, reflective of population distribution. The sample was weighted by gender, age and social class to ensure it was representative. Participants were contacted by Ipsos-MRBI and asked for verbal consent. They were directed to the Ipsos-MRBI website which contained further information about the study and included the study Principal Investigator’s contact details for queries. Participants were not paid for participation.

This study followed STROBE guidelines [20].

### Survey outcomes

Socio-demographic data were collected on age, gender, county of residence, whether respondents had previously been medically diagnosed with COVID-19, and whether they were employed as a healthcare worker, and social class [21].

Other variables, asked in this order, included threat perceptions and fear (ten items), response efficacy and self-efficacy (six items), response cost and social norms (six items), COVID-19 handwashing and physical distancing behaviour (two items), depression (Patient Health Questionnaire-2 [22]), anxiety (Generalised Anxiety Disorder-2 scale [23], and three items on loneliness [24]. All PMT variables were developed based on items described by Williams et al. [25]. Additional items assessed self-reported health, and number of people aged under-18 living in the participant’s household.

### Survey study statistical analysis

We conducted descriptive statistical analyses using the total survey sample and completed an inspection of missing data. There were no missing data. Binary logistic regression models examined sociodemographic and psychological covariates of adherence to handwashing and physical distancing, measured as: *“Over the last two weeks, how often have you been washing your hands frequently for at least 20 s each time?”* and *“Over the last two weeks, how often have you been staying at least 2 m away from others (social distancing)?”* Responses were given on a 4-point Likert Scale (‘1 = Not at all’ to ‘4 = Often’) and recoded as a dichotomous variable. Adherers were classified as those reporting behaviours ‘often’ or ‘sometimes’ and non-adherers classified as those reporting the behaviour ‘rarely’ or ‘not at all’. Multicollinearity was checked prior to modelling and no violations of this assumption were identified. Data analysis was performed using SPSS Version 24.

## Results

### Survey socio-demographics

Table 1 summarises the demographic characteristics of respondents (*n* = 3011). Mean age was 46.7 years (SD: 17.3, age range: 18–97) and 51.2% of respondents were female.

**Table 1:** Weighted demographic characteristics of survey respondents

Demographic characteristics	Number of respondents (%) (total *n* = 3011)
Women	1541 (51.2)
**Household information**	
Mean number of people in the household, including respondent (SD)	3.15 (2.1)
Mean number of people aged under 18 who live in household (SD)	0.67 (1.1)
**Age (years)**	
18–24	333 (11.1)
25–34	536 (17.8)
35–44	598 (19.9)
45–49	219 (7.3)
50–54	289 (9.6)
55–64	457 (15.2)
65–74	387 (12.8)
≥ 75 years	192 (6.4)
**Social class**	
A	136 (4.5)
B	332 (11)
C1	820 (27.2)
C2	615 (20.4)
D	283 (9.4)
E	596 (19.8)
F1	123 (4.1)
F2	37 (1.2)
Refused question	68 (2.3)
**Employed as healthcare worker**	302 (10)
**Previously medically diagnosed with COVID-19**	35 (1.1)
**ROI residence**	
Dublin	664 (29.4)
Rest of Leinster	604 (26.7)
Munster	603 (26.7)
Connaught/Ulster	388 (17.2)
**NI residence**	
Antrim	390 (51.8)
Down	124 (16.5)
Derry/Londonderry	88 (11.7)
Tyrone	67 (8.9)
Armagh	61 (8.1)
Fermanagh	23 (3)

*SD* Standard deviation

The majority of survey non-completion was due to dialling an invalid number (61%), answering machines (10%), no answer (6%) and refusal to participate (3%). The total number and socio-demographics of non-respondents is not available.

### Survey findings

There were no significant changes in responses over the course of the eight-week survey. Median survey responses for all respondents are outlined in Table 2.

**Table 2:** Median response outcomes to psychological variables

Variable	Median	IQR
Depression (1–4, 4 = depressed nearly every day)	1.50	0.5 – 2.5
Anxiety (1–4, 4 = anxious nearly every day)	1.50	0.5 – 2.5
Loneliness (1–3.5, 3.5 = higher loneliness)	1.33	0.3 – 2.3
Self-rated health (1–5, 5 = very bad)	2.0	1—3
Perceived risk of COVID-19 to self (1–5, 5 = high perception of risk)	3.5	2.5 – 4.5
Perceived risk of COVID-19 to others (1–5, 5 = high perception of risk)	4.0	3—5
Perceived vulnerability of self to COVID-19 (1–5, 5 = high perception of vulnerability)	3.0	1.5 – 4.5
Perceived vulnerability of others to COVID-19 (1–5, 5 = high perception of vulnerability)	3.5	2.5 – 4.5
Fear of COVID-19 (1–5, 5 = high fear)	4.0	2–5
Handwashing response efficacy (1–5, 5 = high perception of efficacy)	4.5	3.5 – 5
Social distancing response efficacy (1–5, 5 = high perception of efficacy)	4.5	3.5—5
Handwashing response cost (1–5, 5 = high perception of cost)	1.5	0.5 – 2.5
Social distancing response cost (1–5, 5 = high perception of cost)	2.0	1 – 3
Handwashing self-efficacy (1–5, 5 = high perception of efficacy)	4.0	3 – 5
Social distancing self-efficacy (1–5, 5 = high perception of efficacy)	4.0	2 – 5
Social norms handwashing (1–5, 5 = high perception of social norms)	4.0	3 – 5
Social norms social distancing (5 = high perception of social norms)	4.0	3—5

*IQR* Interquartile range

### Non-adherence with handwashing and social distancing

Most respondents reported adherence to protective behaviours. 2.4% (71 respondents) were non-adherers to handwashing and 6.4% (191 respondents) were social distancing non-adherers.

A binary logistic regression compared handwashing non-adherers and adherers and a separate binary logistic regression analysis compared social distancing non-adherers and adherers (Table 3).

**Table 3:** Binary logistic regression model with non-adherence to handwashing and social distancing as outcomes

	Non-adherence to handwashing				Non-adherence to social distancing			
	*p* value	OR	95% C.I		*p* value	OR	95% C.I	
			Lower	Upper			Lower	Upper
NI (0) vs ROI (1)	.426	.767	.399	1.473	.182	1.332	.875	2.029
Social class B vs A	.190	4.272	.488	37.410	**.022**	**.351**	**.143**	**.860**
Social class C1 vs A	.384	2.580	.305	21.787	**.001**	**.263**	**.118**	**.586**
Social class C2 vs A	.802	1.324	.149	11.783	.593	.816	.387	1.721
Social class D vs A	.542	2.036	.207	20.004	**.021**	**.303**	**.110**	**.835**
Social class E vs A	.335	2.939	.329	26.276	.286	.625	.263	1.484
Social class F1 vs A	.996	.000	.000		.143	.373	.100	1.394
Social class F2 vs A	.524	2.619	.135	50.718	.753	1.287	.267	6.206
Age	.425	.993	.974	1.011	** < .001**	**.975**	**.963**	**.987**
Female (0) vs male (1)	** < .001**	**5.215**	**2.405**	**11.307**	**.011**	**1.672**	**1.123**	**2.487**
Non-hc worker (0) vs hc worker (1)	.631	.684	.146	3.218	**.015**	**1.981**	**1.140**	**3.442**
Depression	.094	.665	.413	1.072	** < .001**	**1.723**	**1.334**	**2.226**
Anxiety	.325	.802	.517	1.245	.186	.836	.642	1.090
Loneliness	**.015**	**1.861**	**1.130**	**3.063**	.537	.903	.652	1.250
No. in household aged under-18	.063	.704	.487	1.019	**.006**	**.746**	**.606**	**.919**
Perceived risk to self	.373	.838	.569	1.235	.244	.877	.702	1.094
Perceived risk to others	.190	1.336	.866	2.061	.597	1.073	.826	1.393
Perceived vulnerability of self	.401	1.176	.806	1.715	.542	.931	.740	1.172
Perceived vulnerability of others	.472	1.149	.787	1.679	.240	.876	.703	1.092
Fear	.116	.801	.608	1.056	**.014**	**.799**	**.667**	**.956**
Response efficacy	.421	.849	.569	1.265	** < .001**	**.570**	**.454**	**.716**
Response cost	** < .001**	**3.401**	**2.233**	**5.180**	**.031**	**1.309**	**1.026**	**1.670**
Social norms	** < .001**	**.481**	**.324**	**.714**	**.003**	**.728**	**.592**	**.895**
Self-efficacy	** < .001**	**.486**	**.376**	**.628**	** < .001**	**.668**	**.571**	**.783**

*OR* Odds Ratio, *CI* Confidence interval, *hc* healthcare

Results highlighted in bold indicate statistically significant (*p* < 0.05) covariates

Handwashing non-adherers were more likely to be male, to have higher levels of loneliness, higher perceptions of handwashing costs, lower perceptions of self-efficacy of handwashing and lower perceptions of handwashing social norms.

Social distancing non-adherers were more likely to be in lower social class groups, to be younger, male, healthcare workers, have lower mood, fewer people living in household under-18, lower fear, lower perceptions of the response efficacy of distancing, lower self-efficacy in social distancing, lower social norms in social distancing, and higher response cost.

No differences were observed in social distancing or handwashing non-adherence between residents in NI or ROI.

## Discussion

The effectiveness of public health measures like handwashing and social distancing is dependent on the general public’s adherence. Most of the general public surveyed in this study reported themselves as adhering to guidelines.

While there is some overlap (male gender, high response cost, low self-efficacy, low social norms), handwashing and distancing non-adherers’ profiles represent two distinct groups. Social distancing non-adherers are more likely to be health care workers and may be unable to social distance in healthcare settings, rather than unwilling to. Similarly, those in lower, middle and working class social groups likely live in poorer quality, higher density housing with fewer opportunities to distance from the community [26]. Some distancing non-adherers may be reporting inability to distance, rather than a lack of desire to do so.

That male gender predicts non-adherence to both behaviours in our findings aligns with the rich literature on men’s increased propensity to engage in risk behaviours compared to women [18, 27] and has also been reported in a recent PMT survey predicting protective behaviours during the COVID-19 pandemic [17]. This finding isn’t reflected within COVID-19 epidemiological data as COVID-19 prevalence is the same in men and women [28]. Nonetheless, that men are more likely to non-adhere is a concern as they have higher risk of COVID-19 complications and death [28]. Specific ways in which to communicate a health message to men directly are required.

We found no differences in non-adherence and PMT variables in respondents living in NI and ROI. This suggests both populations are broadly compliant, despite some differences between jurisdictions in implementation of public health measures.

Respondents with fewer children living in the household were more likely to report non-adherence to distancing. Adults with children may be more likely to practice distancing to protect their children. Alternatively, parents and guardians may have more opportunities to social distance as they remain at home to care for and educate children. Not living with children will be associated with younger age, but our analysis showed younger age as an independent covariate of social distancing non-adherence.

Our findings suggest younger adults are more likely not to social distance, a consistent finding in emerging COVID-19 literature [18] and echoing recent statements by the World Health Organization who warned asymptomatic people in their 20 s and 30 s have driven the viral spread. Framing public health messaging towards younger people in a bid to increase their sense of social norms, encouraging their self-efficacy and response efficacy while promoting the use of remote technology to stay connected, could be effective means of lowering transmission of the virus.

Our findings both align and diverge with previous adherence behaviour research. Loneliness has been linked to health promoting behaviours, the rationale being that compliance with social distancing can increase loneliness [29]. Our results found no link between loneliness and distancing non-adherence, however, handwashing non-adherers had higher loneliness, similar to recent findings elsewhere [30]. Those who experience loneliness may be more likely to live alone and may perceive themselves as less at risk of infection via the touching of objects and surfaces.

Previous research links low mood to medical treatment non-adherence [31] and anxiety to higher health behaviour adherence [32]. However, Wright et al. [30] found little evidence of any association between mental health and adherence, arguing anxiety and depression “*have multiple, countervailing effects on compliance…meaning the net association is context specific*”. We found no links between mood variables and handwashing non-adherence; however, distancing non-adherers were more likely to have low mood. Those with low mood may lack motivation or desire to engage in the protective behaviour. The effect of knowingly not adhering to distancing guidelines when most of the population report compliance may instil low mood. Alternatively, people who value and seek social engagement may be more willing to contravene distancing guidelines and more susceptible to lower mood if others withhold contact.

There were no links between perceptions of vulnerability or risk and non-adherence. Lower COVID-19 fear did predict distancing non-adherence, suggesting some form of risk perception is linked to non-adherence. That this result was independent of age and male gender, suggests a robust finding. Our finding on COVID-19 fear echoes previous research which found feeling personally at risk of infection predicted greater propensity to engage in social distancing in the early stages of the pandemic [33]. COVID-19 protective behaviours has consistently found fears, threat appraisals and worries are linked to increased adherence [15, 34, 35] and that fear is a stronger predictor of adherence than moral or political orientation [36], although an Iranian online survey reported increased fear predicted less adherence [13]. With the exception of Rad et al.’s survey [13], these findings are in line with PMT theory which predicts that fears concerning events or outcomes can facilitate behavioural change [37]. The finding on COVID-19 fear in this study and others suggests clear communication of risk could improve engagement in protective behaviours, particularly when targeting disengaged groups.

Our finding that types of non-adherence have different demographic and psychological covariates is novel and indicates that public health messages should be tailored to the specific behaviour of interest as well as to specific populations, rather than generic calls for compliance. Distancing non-adherence has emotional and social underpinnings like low fear, mood, social class and is associated with the people we share homes with and the places we work. While handwashing non-adherence appears to have fewer emotional and mood components, both behaviours share some predictive variables, including male gender, perceptions of response cost, social norms and self-efficacy. Public health campaigns could improve effectiveness if they emphasise the severity of the virus, the efficacy of protective behaviours and the socially normative elements of these behaviours. Our findings suggest that focusing on individuals’ and others’ risk and vulnerabilities to COVID-19 will not influence behaviour. A future study could specify the effect of who we perceive ‘others’ to be on our behaviours, for example, people may indicate more adherence if others are family members or friends, rather than a more generic general public.

The analyses suggest some differential factors should be considered to effect change in social distancing. Fear predicts social distancing; this needs to be carefully balanced against a possible mental health impact. Public health messaging could also increase awareness of the effectiveness of social distancing measures, to increase response efficacy. These suggestions are tentative as our data is observational. To have greater confidence in the direction of variable influence, an experimental study could quantify adherence intention following engagement with public health messaging designed to alter the aforementioned factors.

This study has several strengths. The use of convenience and non-probability samples in COVID-19 research has been widespread due to the push for rapid information, but these methods are at risk of substantial bias [38]. This study is one of the largest cross-sectional studies of its kind and unlike convenience sampling common in online surveys, this survey has been weighted for sex, age and socio-economic status. The use of a telephone survey method means results are not biased towards those with internet access. The survey began in early June 2020, three months after the first case on the island of Ireland and after the imposition of a widespread lockdown. Respondents’ cognitions and beliefs regarding the pandemic and the appropriate behavioural response were likely well-established. Finally, this study has a novel design in that it incorporates two jurisdictions with two separate public health systems, which occupy one land mass. It is the only current survey representative of both parts of Ireland. The finding that no differences in behaviour emerged between populations suggests adherence behaviours are more similar than different, and cultural or macro-level factors may play less of a role than demographic and psychological factors.

This study has several limitations. Most respondents reported behavioural adherence and as a result, the number of non-adherers to either behaviour was small. There may be biases due to self-report. Due to social desirability concerns, the proportion of those reporting non-adherence is likely an underestimate of the true figure. This study was explicitly introduced by recruiters as a study investigating COVID-19 opinions on the island of Ireland. Those who agreed to answer such questions may be more interested in COVID-19 generally and more knowledgeable of the guidelines, however the study had a very low refusal rate. The survey was carried out during a period when COVID incidence and mortality had reduced and lockdown measures had eased. The high reported compliance may be an artefact of this easing. The survey was conducted during the summer months of 2020 when social distancing was easier due to better weather. We would expect some variation in findings had this survey run during winter or a more severe lockdown. In addition, this research was conducted during a specific time period in the context of an evolving pandemic situation. Although that does not diminish the value of the findings, there will be other public health priorities that have developed that are not part of this research (eg. vaccine uptake) . 

## Conclusions

Overall, we found that a PMT model is effective in differentiating between groups of non-adherers. While we found most respondents surveyed were adhering to social distancing and handwashing advice, it is important to understand differences in behaviours driven by groups in society that report not routinely adhering to guidelines.

Our findings provide important information for the design of public health messages that intend to encourage handwashing and social distancing for the purposes of minimising spread of a virus. We suggest that resources should be directed to allow these public health messages to reach younger people and men, in particular. Public health messaging should be framed in a way that increases people’s beliefs that by engaging in these behaviours they are following the social norms, increases self-efficacy about engaging in the behaviours, and highlights the beneficial effects of the behaviours.We believe that this strategy will enhance adherence to two of the most important preventive health behaviours in relation to reducing the community transmission of COVID-19 and similar diseases.

## Data Availability

Deidentified participant data are available from Catherine Darker (catherine.darker@tcd.ie) upon reasonable request and for the purposes of public health planning or scientific research.

## Abbreviations

CI: Confidence interval
COVID-19: Coronavirus Disease 2019
HC: Healthcare
NI: Northern Ireland
OR: Odds ratio
PMT: Protection Motivation Theory
SARS: Severe Acute Respiratory Syndrome
SD: Standard deviation
STROBE: Strengthening the Reporting of Observational Studies in Epidemiology
ROI: Republic of Ireland
UK: United Kingdom
